# Gastrotomy incision to Bogotá bag suturing (GIBBS) technique for trichobezoar extraction

**DOI:** 10.1308/rcsann.2025.0115

**Published:** 2026-03-05

**Authors:** M Mardan, Y-W. Tan

**Affiliations:** University of Malaya, Kuala Lumpur, Malaysia

Gastric trichobezoar extraction through a gastrotomy carries significant risk of peritoneal contamination and surgical site infection (SSI) of 6–14%, because intraoperative handling of the hairball inadvertently squeezes trapped filthy fluid.^1–4^

A 14-year-old girl presented with 2 days of bilious vomiting, abdominal distension and pain. She was dehydrated with hyponatraemia. Computed tomography showed a gastric trichobezoar extending into the second part of the duodenum and a concomitant obstructive satellite bezoar at mid-ileum (Figure 1).

**Figure 1:**
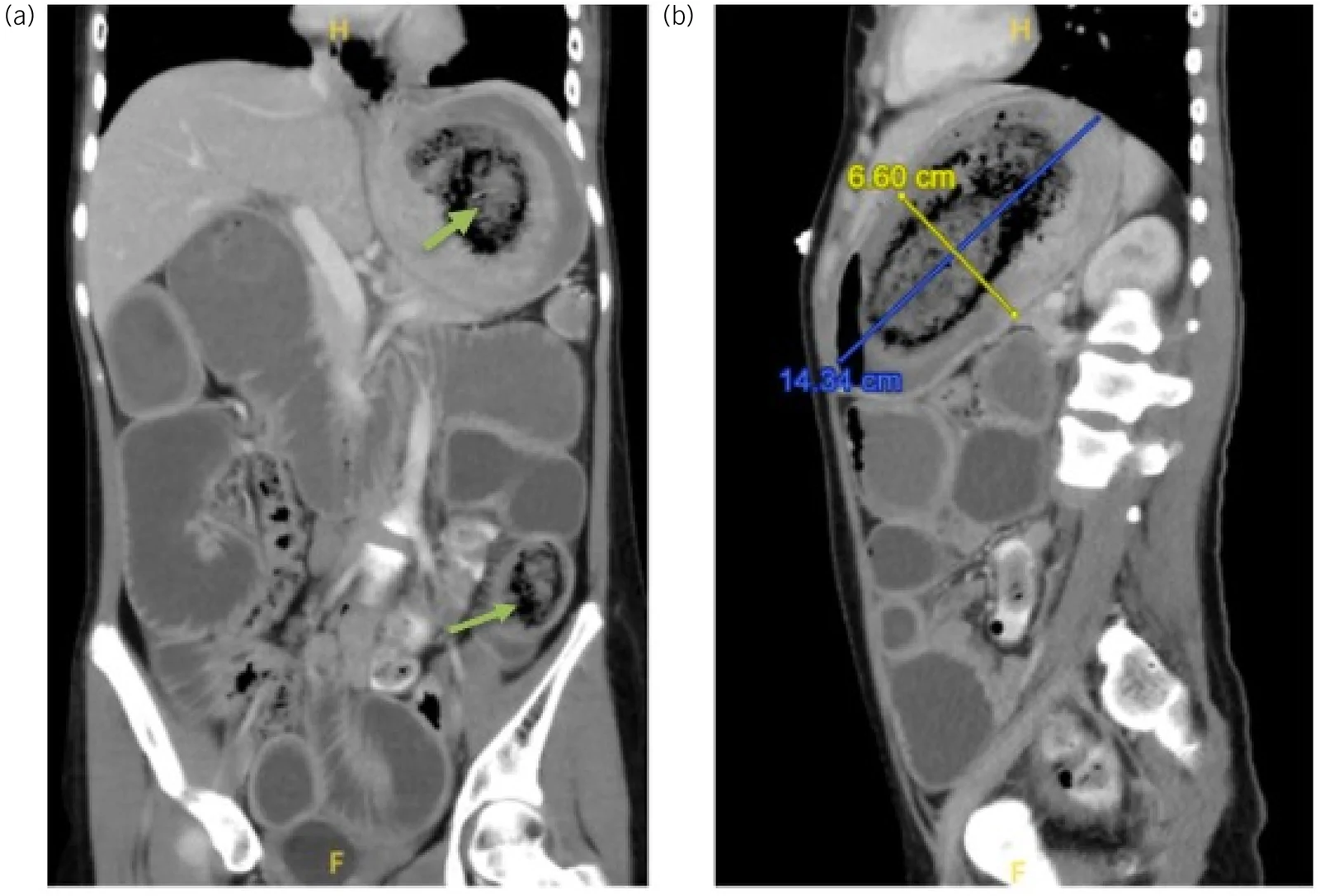
(a) Coronal computed tomography (CT) view showing a large intragastric trichobezoar (short arrow) and a satellite bezoar in the small bowel (long arrow). (b) Sagittal CT view with measurements showing the bezoar spanning approximately 14.3cm in length and 6.6cm in width

Through an upper-midline laparotomy, an 8cm gastrotomy was made along the long-axis of the anterior stomach, which was sutured full-thickness, using 2/0 polyglactin, to the edges of a cut-out hole at the centre of a Bogotá bag, approximately the size of the gastric incision. The Bogotá bag was exteriorised extracorporeally to achieve peritoneal exclusion (Figure 2). The bezoar was reorientated with its ‘fundus’ towards the gastrotomy for extraction with Allis forceps (Figure 3). Retrograde milking of the ileal satellite bezoar into the gastrotomy negated an enterotomy. Finally, irrigation of the lumen was achieved before the removal of Bogotá bag, followed by a two-layer gastric closure.

**Figure 2:**
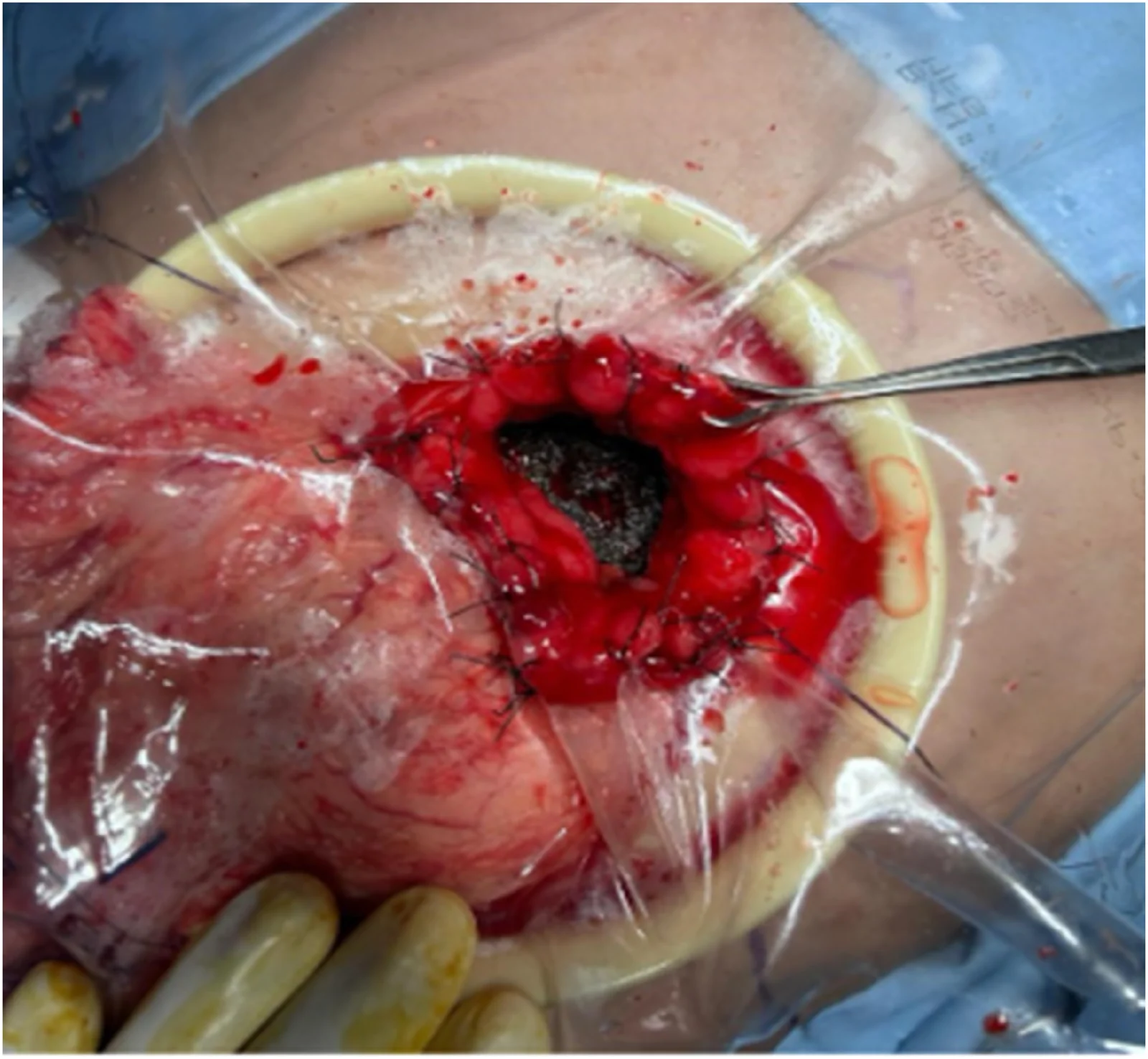
Exteriorised Bogotá bag with a hole sutured circumferentially to the gastrotomy for peritoneal exclusion

**Figure 3:**
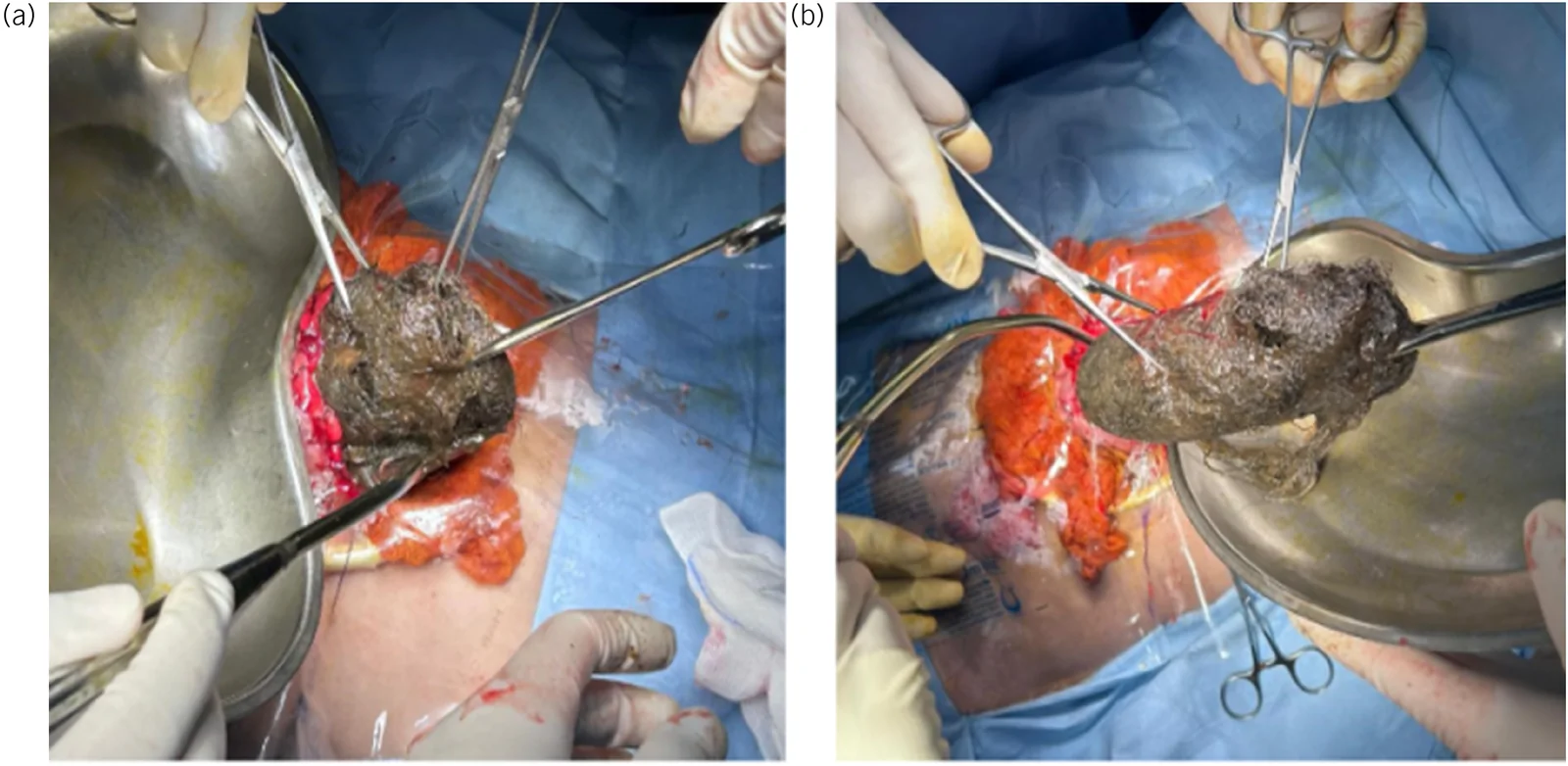
(a) Extraction by orientating the fundus of the bezoar into the gastrotomy. (b) Complete extraction of the intact bezoar without peritoneal contamination using the gastrotomy incision to Bogotá bag suturing technique

The gastrotomy incision to Bogotá bag suturing (GIBBS) technique offers an excellent seal. With the confidence of spillage mitigation, bezoar extraction through a modest gastric incision becomes achievable with adequate compression and strategic orientation of the mass. It converts a contaminated surgery into a clean-contaminated one. We have successfully deployed GIBBS in three cases of Rapunzel syndrome without SSI. This technique may offer comparable benefits in extraction of foreign body from other hollow viscera.

## Competing interests

The author/s declare no competing interests.

## Funding

The author/s received no financial support for the research, authorship and/or publication of this article.

## Ethics statement

Informed consent was obtained from the patient’s guardian.

## Consent to participate statement

Not applicable.

## Author contributions

**Y-W Tan**: Conceptualisation, Data curation, Formal analysis, Investigation, Project administration, Resources, Supervision, Validation, Visualisation, Writing – review & editing. **M Mardan**: Data curation, Formal analysis, Investigation, Project administration, Validation, Visualisation, Writing – original draft, Writing – review & editing.

## Artificial Intelligence

The author/s declare that no AI was used to conduct the study or prepare the manuscript.
